# Metabolomic profile, anti-trypanosomal potential and molecular docking studies of *Thunbergia grandifolia*

**DOI:** 10.1080/14756366.2023.2199950

**Published:** 2023-04-20

**Authors:** Heba A. S. El-Nashar, Ahmed M. Sayed, Hany A. M. El-Sherief, Mostafa E. Rateb, Lina Akil, Ibrahim Khadra, Taghreed A. Majrashi, Sara T. Al-Rashood, Faizah A. Binjubair, Mahmoud A. El Hassab, Wagdy M. Eldehna, Usama Ramadan Abdelmohsen, Nada M. Mostafa

**Affiliations:** a Department of Pharmacognosy, Faculty of Pharmacy, Ain Shams University, Cairo, Egypt; b Department of Pharmacognosy, Faculty of Pharmacy, Nahda University, Beni-Suef, Egypt; c Department of Pharmaceutical Chemistry, Faculty of Pharmacy, Deraya University, Minia, Egypt; d School, of Computing, Engineering & Physical Sciences, University of the West of Scotland, Paisley, UK; e Strathclyde Institute of Pharmacy & Biomedical Sciences, University of Strathclyde, Glasgow, UK; f Department of Pharmacognosy, College of Pharmacy, King Khalid University, Abha, Saudi Arabia; g Department of Pharmaceutical Chemistry, College of Pharmacy, King Saud University, Riyadh, Saudi Arabia; h Department of Medicinal Chemistry, Faculty of Pharmacy, King Salman International University (KSIU), Ras Sudr, Egypt; i Department of Pharmaceutical Chemistry, Faculty of Pharmacy, Kafrelsheikh University, Kafrelsheikh, Egypt; j Department of Pharmacognosy, Faculty of Pharmacy, Minia University, Minia, Egypt; k Department of Pharmacognosy, Faculty of Pharmacy, Deraya University, Universities Zone, New Minia City, Egypt

**Keywords:** *Trypanosoma brucei*, *Thunbergia grandifolia*, LC-MS, *in silico*, inverse docking

## Abstract

Trypanosomiasis is a protozoan disease transmitted via* Trypanosoma brucei.* This study aimed to examine the metabolic profile and anti-trypanosomal effect of methanol extract of *Thunbergia grandifolia* leaves. The liquid chromatography-high resolution electrospray ionisation mass spectrometry (LC-HRESIMS) revealed the identification of fifteen compounds of iridoid, flavonoid, lignan, phenolic acid, and alkaloid classes. The extract displayed a promising inhibitory activity against *T. brucei* TC 221 with MIC value of 1.90 μg/mL within 72 h. A subsequent in silico analysis of the dereplicated compounds (i.e. inverse docking, molecular dynamic simulation, and absolute binding free energy) suggested both rhodesain and farnesyl diphosphate synthase as probable targets for two compounds among those dereplicated ones in the plant extract (i.e. diphyllin and avacennone B). The absorption, distribution, metabolism, excretion, and toxicity (ADMET) profiling of diphyllin and avacennone were calculated accordingly, where both compounds showed acceptable drug-like properties. This study highlighted the antiparasitic potential of *T. grandifolia* leaves.

## Graphical Abstract

**Figure:**
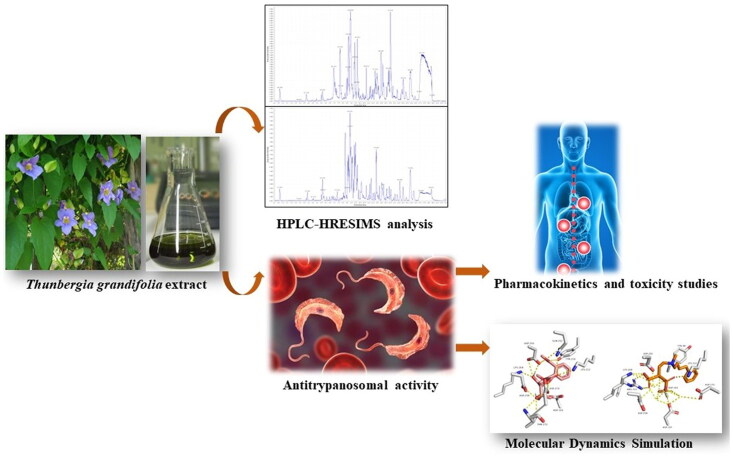

## Introduction

Trypanosomiasis or sleeping sickness is a protozoan disease that infects animals and humans transmitted by the bite of *Glossina* (tsetse) fly carrying *Trypanosoma brucei*^1^. Currently, trypanosomiasis affects more than 50 million cattle and 70 million people in sub-Saharan Africa^2^. The available current medicines record lack of efficiency, resistance, and toxicity, so there is an urgent need for the development of novel, safe, efficacious, cost-effective drugs with new mechanism of action^3^^,^^4^. In African countries where trypanosomiasis is prevalent, natural products (herbal extracts) have traditionally been utilised for centuries and are still extensively used to cure infections and other parasitic diseases^5^^,^^6^. Interestingly, about 30% of the world population has confidence in traditional therapies due to their wide availability and affordability^7^. Moreover, various drugs like quinine and artemisinin were established as plant-derived potential antiprotozoal agents^8^.

*Thunbergia* is a dicotyledonous flowering genus, belonging to the family Acanthaceae consisting of more than 100 species^9^. The plants of this species are climbers, shrubs, perennials, and annuals distributed in tropical regions^10^. Some of the plants in the *Thunbergia* genus are well-known for their medicinal properties and ornamental value, such as *Thunbergia laurifolia*, *Thunbergia alata*, *Thunbergia erecta*, *Thunbergia coccinea*, *Thunbergia colpifera,* and *Thunbergia fragrans*^11^. Among these plants*, T. laurifolia* was the first to be consumed widely for human consumption in both traditional and local preparations^12^. The literature survey revealed that *Thunbergia* plants are rich in phytoconstituents, like iridoids, tannins, phenolic acids, flavonoids, and their glucosides^9^^,^^13^. Besides, these constituents are reported to exert several biological potentials such as antioxidant, anti-inflammatory, hepatoprotective, antinociceptive, antipyretic, antitumor, antimicrobial, antidiabetic, and anthelmintic activities^14–17^. Among *Thunbergia* species, *Thunbergia grandiflora* Roxb, known as Nallata is a large perennial, hard, climbing or twining plant (up to 15 m) with blue flowers^9^^,^^18^. It is widely distributed in India, China, Myanmar, and several tropical countries of Africa especially Egypt and Bangladesh^19^.

In traditional medicine, *T. grandiflora* was reported to manage several ailments like blood dysentery, cataract, diabetes, gout, hydrocele, hysteria, malaria, marasmus, post-eclampsia, pre-eclampsia, rheumatism, spermatorrhoea, stomach ache, ophthalmia, conjunctivitis, elephantiasis, and urinary bladder stone^20^. Pharmacologically, it exerted valuable biological properties including antimicrobial^21^, anti-inflammatory and anti-arthritic effects^22^. From the phytochemical view, *T. grandiflora* contains important phytochemicals as iridoid glycosides, including isounedoside and grandifloric acid, in addition to flavonoids as malvidin-3,5-diglucoside, 5-hydroxy-4′,6,7-trimethoxyflavone, luteolin-7-glucoside, apigenin-7-glucuronide, stilbericoside, proanthocyanidin, and the aglycone luteolin^23^^,^^24^.

Due to its valuable secondary metabolites content and plentiful pharmacological and ethnobotanical survey of *T. grandiflora*, the current study was undertaken to examine the metabolic profile and anti-trypanosomal effects of the methanolic leaf extract of *T. grandifolia*. Furthermore, *in-silico* docking studies were carried out to illustrate the mechanism of action of identified secondary metabolites.

## Materials and methods

### Plant material

The fresh leaves of *T. grandifolia* were collected from Zoo Garden, Giza, Egypt (30°1′28.32″N 31°12′50.03″E) in February 2021. The plant was taxonomically identified by Mrs. Tereize Labib, the taxonomy specialist at El-Orman Botanical Garden, Giza, Egypt. A voucher specimen (PHG-P-TG-365) has been kept in the Herbarium of the Pharmacognosy Department, Faculty of Pharmacy, Ain Shams University, Cairo, Egypt.

### Preparation of plant extract

The fresh aerial parts of *T. grandifolia* (1 kg) were exhaustively extracted with absolute methanol (9.5 L) by percolation at room temperature until depletion. Then, the extract was filtrated and concentrated under reduced pressure using rotavapor at 45 °C to yield 30 g of completely dry extract.

### Metabolic profile analysis conditions

The crude extract (1 mg/mL) in methanol (MeOH) was subjected to metabolic analysis using LC-HR-ESI-MS according to the previously reported method^25^. Acquity Ultra Performance Liquid Chromatography (UPLC) system coupled to a Synapt G2 HDMS quadrupole time-of-flight hybrid mass spectrometer (Waters, Milford, USA) was used. Chromatographic separation was carried out on a BEH C18 column (2.1 × 100 mm, 1.7 μm particle size; Waters, Milford, USA) with a guard column (2.1 × 5 mm, 1.7 μm particle size) and a linear binary solvent gradient of 0–100% eluent B over 6 min at a flow rate of 0.3 ml/min, using 0.1% formic acid in water (v/v) as solvent A and acetonitrile as solvent B. The injection volume was 2 μL and the column temperature was 40 °C. The total analysis time for each sample was 20 min. High-resolution mass spectrometry was carried out in both positive and negative ESI ionisation modes coupled with a spray voltage at 4.5 kV, capillary temperature at 320 °C, and mass range from *m/z* 150–1500. The MS dataset was processed, and data were extracted using MZmine 2.20 based on the established parameters^26^^,^^27^. Mass ion peaks were detected and accompanied by chromatogram builder and chromatogram deconvolution. The local minimum search algorithm was addressed, and isotopes were also distinguished via the isotopic peak grouper. Missing peaks were displayed using the gap-filling peak finder. An adduct search along with complex search were done. The processed data set was next subjected to molecular formula prediction and peak identification. The positive and negative ionisation mode data sets from the respective extract were dereplicated against the DNP (Dictionary of Natural products).

### Investigation of anti-trypanosomal activity

The anti-trypanosomal activity was tested following the protocol of^28^. Briefly, 10^4^ trypanosomes per ml of *T. brucei brucei* strain TC 221 were cultivated in Complete Baltz Medium. Trypanosomes were tested in 96-well plate chambers against different concentrations of test extracts at 10–200 μg/mL in 1% DMSO to a final volume of 200 μL. For controls, 1% DMSO as well as parasites without any test extract was used simultaneously in each plate to show no effect of 1% DMSO. The plates were then incubated at 37 °C in an atmosphere of 5% CO_2_ for 24 h using a CO_2_ incubator (CelMate^®^, ESCO™, Singapore). After the addition of 20 μL of Alamar Blue, the activity was measured after 48 and 72 h by light absorption using an MR 700 Microplate Reader at a wavelength of 550 nm with a reference wavelength of 650 nm. The minimum inhibitory concentration (MIC) values of the test extracts were quantified in by linear interpolation of three independent measurements. Suramin was used as a positive control (MIC = 0.23 μg/mL).

### *In silico* study

Both inverse docking, molecular dynamics simulation, and absolute binding free energy calculation were carried out according to the previously reported methods^29^^,^^30^. The detailed methodology can be found in the Supplementary File.

## Results and discussion

### Analysis of metabolic profile

As shown in Figure S1, LC-HRESIMS metabolic profiling of *T. grandifolia* resulted in the identification of fifteen compounds of various classes of plant metabolites. The identified compounds are listed in Table 1. These compounds are identified as five iridoid glucosides (stilbericoside, alatoside, 5-deoxythunbergioside, thunaloside, and isounedoside), three napthoquinones (avicennone B, 4-deoxyavicennone B and 3a-deoxyavicennone G) and two flavonoids (3,5,7-trihydroxy-3′,4′-dimethoxyflavone and 5,7-dihydroxy-4′-methoxyflavone). In addition, lignan (diphyllin), one alkaloid (aphelandrine) and one fatty acid (6-hexadecenoic acid) were also characterised.

**Table 1.** LC-HRESIMS-dereplicated phytochemicals in the methanol extract of *Thunbergia grandifolia.*

No.	R_t_ (min)	*m/z*	Ionisation mode	Calculated mass	Accurate mass	Molecular formula	Putative identification	Chemical class
1.	0.63	379.08225	Negative	380.08953	380.089605	C_21_H_16_O_7_	Diphyllin	Lignans
2.	3.40	254.22458	Positive	253.22458	254.224580	C_16_H_30_O_2_	6-Hexadecenoic acid	Fatty acid
3.	4.44	331.08196	Positive	330.07468	330.073955	C_17_H_14_O_7_	3,5,7-Trihydroxy-3′,4′-dimethoxyflavone	Flavonoids
4.	4.45	719.15891	Positive	718.15163	718.153390	C_36_H_30_O_16_	Rosmarinic acid dimer	Phenolic acids
5.	4.51	337.12848	Positive	336.1212	336.120905	C_17_H_20_O_7_	Avicennone B	Napthoquinones
6.	4.57	349.09262	Positive	348.10565	348.105649	C_14_H_20_O_10_	Stilbericoside	Iridoid glucosides
7.	5.34	333.10983	Negative	334.11711	334.126385	C_14_H_22_O_9_	Alatoside	Iridoid glucosides
8.	6.03	319.11782	Negative	320.1251	320.125990	C_17_H_20_O_6_	4-Deoxyavicennone B	Napthoquinones
9.	6.77	369.09494	Positive	368.08766	368.087412	C_14_H_21_ClO_9_	5-Deoxythunbergioside	Iridoid glucosides
10.	6.80	229.08642	Positive	228.07914	228.078645	C_14_H_12_O_3_	Naphtho[2,3-b]furan-4,9-diol; di-Me ether	Napthoquinones
11.	7.78	491.26569	Negative	492.27297	492.273656	C_28_H_36_N_4_O_4_	(+)-Aphelandrine	Alkaloids
12.	8.15	349.19842	Positive	348.14204	348.142035	C_15_H_24_O_9_	Thunaloside	Iridoid glucosides
13.	9.48	263.12852	Positive	262.12124	262.120509	C_15_H_18_O_4_	3a-Deoxyavicennone G	Napthoquinones
14.	9.51	285.07621	Positive	284.06894	284.068475	C_16_H_12_O_5_	5,7-Dihydroxy-4′-methoxyflavone	Flavonoids
15.	10.43	331.1905	Negative	332.11764	332.110735	C_14_H_20_O_9_	Isounedoside	Iridoid glucosides

*Note:* Rt: retention time; m/z: mass-to-charge ratio.

The iridoid glucosides represent the major components of the extract. The mass ion peak at *m/z* 349.09262 [M + H]^−^ for the predicted molecular formula C_14_H_20_O_10_ was dereplicated as stilbericoside which was formerly characterised from *T. alata*^31^, whereas that at *m/z* 333.10983 [M − H]^−^ for the suggested molecular formula C_14_H_22_O_9_ was dereplicated as alatoside. This compound was also isolated previously from *T. alata* and *Thunbergia coccinea*^32^. Moreover, the mass ion peak at *m/z* 369.09494 [M + H]^−^, corresponding to the predicted molecular formula C_14_H_21_ClO_9_, was identified as 5-deoxythunbergioside, earlier obtained from *Odontonema cuspidatum* (Acanthaceae)^33^. Another mass ion peak at *m/z* 349.19842 [M + H]^−^ in agreement with the molecular formula C_15_H_24_O_9_ was characterised as thunaloside that was previously identified from *T. alata*^31^. Likewise, the mass ion peak at *m/z* 331.1905 [M − H]^−^ for the predicted molecular formula C_14_H_20_O_9_ was dereplicated as isounedoside. The latter is a metabolite formerly reported from *T. alata*^34^.

Regarding identified flavonoids, the mass ion peak at *m/z* 331.08196 [M + H]^−^, in accordance with the molecular formula C_17_H_14_O_7_, was dereplicated as 3,5,7-trihydroxy-3′,4′-dimethoxyflavone previously isolated from *Andrographis viscuosula* (Acanthaceae)^35^, while that at *m/z* 285.07621 [M–H]^−^, corresponding to the molecular formula C_16_H_12_O_5_, was identified as 5,7-dihydroxy-4′-methoxyflavone that was also reported from *T. laurifolia*^36^.

**Figure 1.**
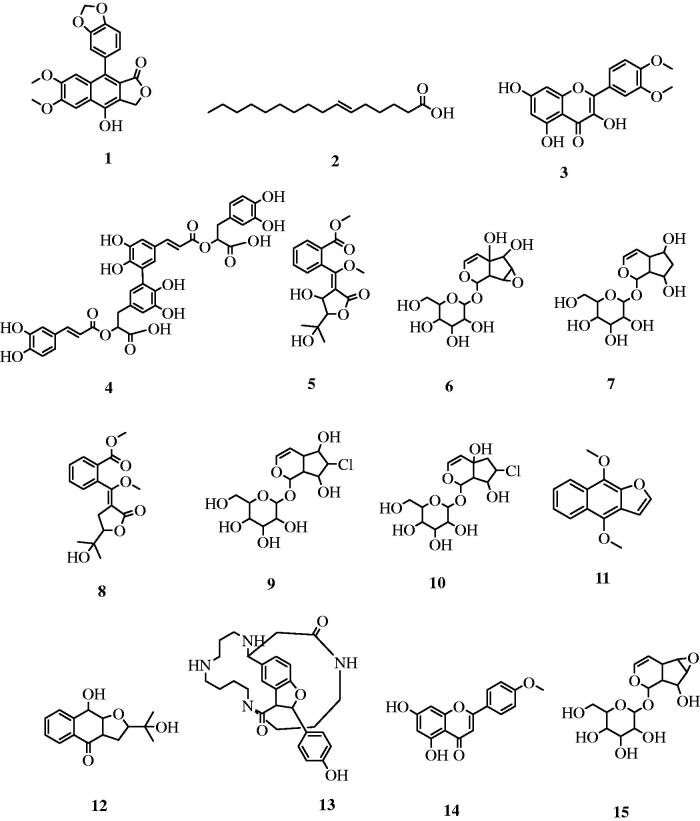
Chemical structures of identified compounds in the methanol extract of *T. grandifolia.*

Furthermore, the mass ion peak at *m/z* 379.08225 [M − H]^−^, following the molecular formula C_21_H_16_O_7_, was dereplicated as diphyllin previously isolated from *Justicia gendarussa* (Acanthaceae)^37^. The alkaloid at *m/z* 491.26569 [M − H]^−^, corresponding to the molecular formula C_28_H_36_N_4_O_4_, was identified as (+)-aphelandrine that was also reported from *Aphelandra tetragona* that belongs to the family Acanthaceae^38^. The mass ion peak at *m/z* 254.224580 [M + H]^−^, for the suggested molecular formula C_16_H_30_O_2_ was dereplicated as 6-hexadecenoic acid^39^, a fatty acid previously obtained from *T. alata* seeds^40^. Finally, rosemarinic acid was reported in the aqueous methanolic extract of *T. erecta* and *T. laurifolia*^10^^,^^41^. The chemical structures of characterised metabolites (**1–15**) are illustrated in Figure 1.

### Anti-trypanosomal activity

The effective chemotherapeutic agents in the treatment of trypanosomiasis are still in great demand^42^. The available drugs such as uramin and pentamidine, are only effective against the early blood stage infection of *T. brucei rhodesiense*^43^. Furthermore, the drugs that maybe effective against the West African sleeping sickness caused by *T. brucei gambiense* may not be efficient against *T. brucei rhodesiense*^44^. Thus, our study underlines the necessity to peek into medicinal plants for drug discovery. In our study, the extract showed a promising inhibitory activity against *T. brucei* TC 221 with MIC value of 1.90 μg/mL within 72 h, thereby confirming presence of anti-trypanosomal compounds in the plant. The HPLC-MS analysis reported presence of iridoid glycosides, napthoquinones, lignans and flavonoids as shown in Table 1. It may be possible that the extract exerted the anti-trypanosomal action with iridoid glycosides as has been previously declared against *Trypanosoma*^45–47^. Furthermore, flavonoids and lignans of *Virola surinamensis* twigs were reported for activity against trypomastigote form of *Trypanosoma cruzi*^48^.

### *In silico* investigation

#### Inverse docking

*In silico* analysis of the studied extract was achieved by subjecting the structures of all dereplicated compounds to inverse docking-based virtual screening against almost all protein structures hosted in the Protein Data Bank (PDB)^49^^,^^50^.

This preliminary virtual screening step was accomplished using idTarget online platform^51^. The recovered scores were obtained as a list, beginning with the largest negative value, and ending with the smallest. To identify the best targets for each isolated compound, we used a conclusive affinity score of −9 kcal/mol as a cut-off value.

Intriguingly, between all mentioned molecular targets, rhodesain protease, and farnesyl diphosphate synthase targets were detected between these target compounds. These enzymes are responsible for trypanosome survival activity^52^^,^^53^, so the preliminary virtual screening step putatively identified these compounds as probable anti-trypanosomal agents.

**Figure 2.**
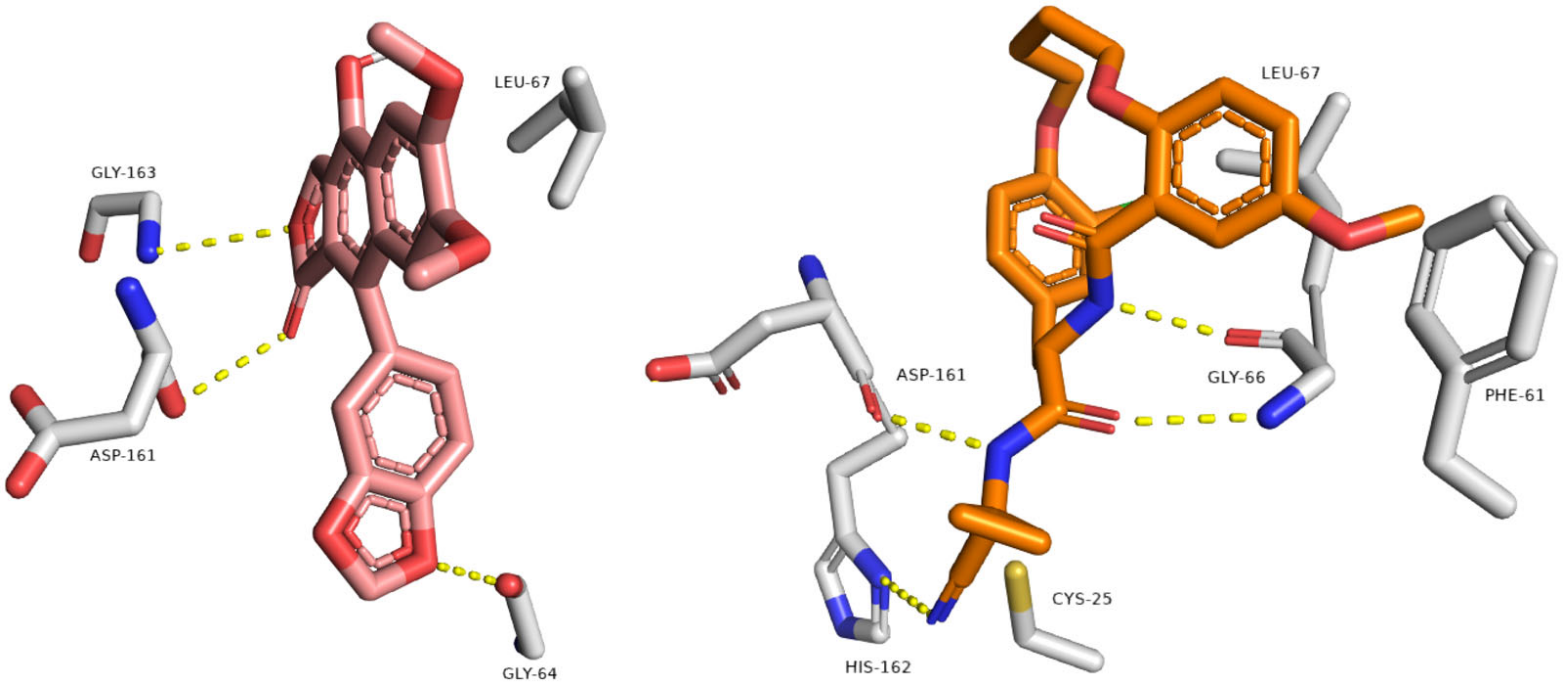
Binding modes of diphyllin (left side) inside the active site of rhodesain target. Binding mode of co-crystalized ligand (right side) inside the active site of rhodesain.

Figure 2 shows remarkable binding mode network of diphyllin with its molecular target rhodesain. Binding mode similarity of diphyllin with co-crystallized ligand of rhodesain rationalised its potent anti-trypanosomal activity. Where co-crystallized rhodesain ligand (6exq) and diphyllin showed H-bond framework with different amino acids such as GLY-163, ASP-161, GLY-64, CYS25, ASP161, GLY66, and ASP60.

**Figure 3.**
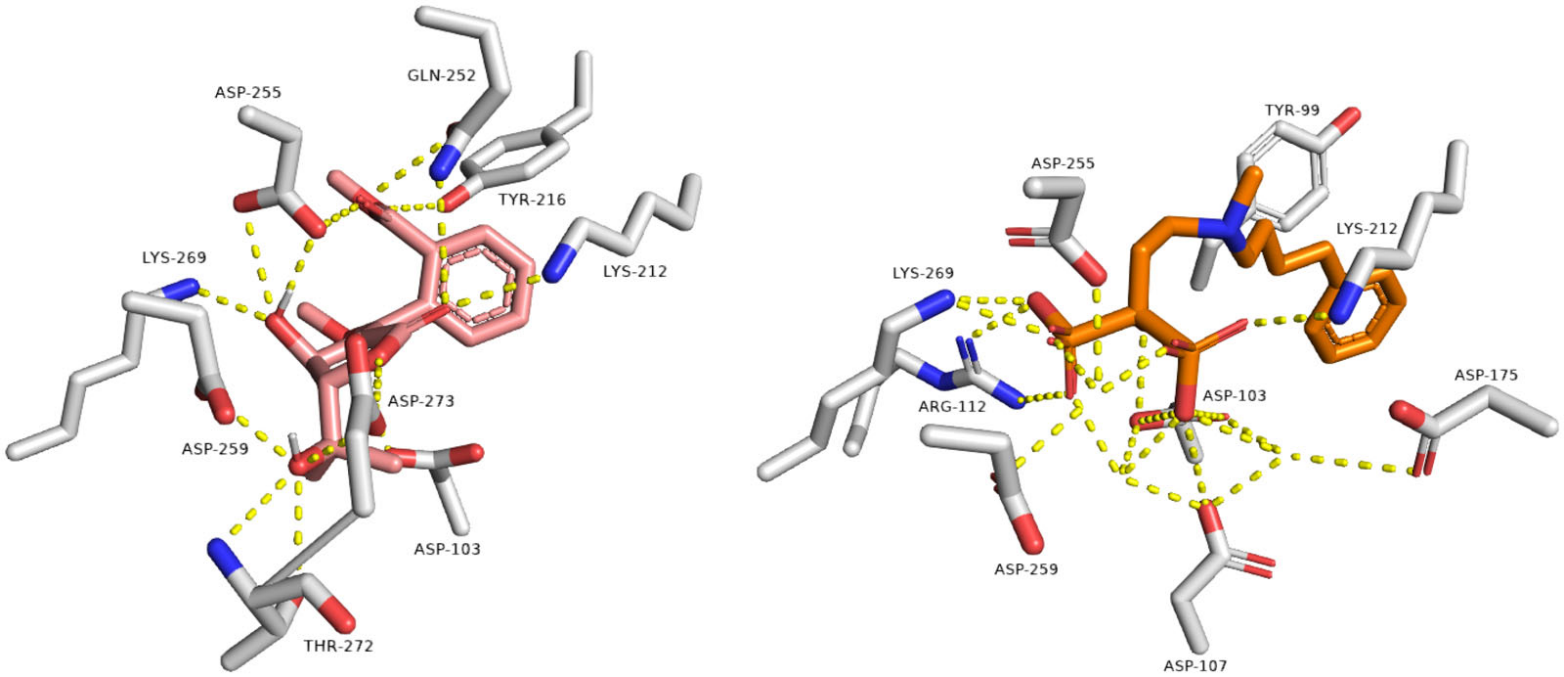
Binding modes of avicennone B (left side) inside the active site of farnesyl diphosphate synthase target. Binding mode of co-crystalized ligand (right side) inside the active site of farnesyl diphosphate synthase.

Regarding farnesyl diphosphate synthase target, compound avicennone B showed significant binding inside its active site through H-bond network that it established with THR272, ASP259, ASP255, GLN252, TYR216, and LYS269 through different functional groups as hydroxyl and carbonyl groups (Figure 3). Moreover, farnesyl diphosphate synthase co-crystallized ligand showed characteristic binding mode with different hydrogen and hydrophobic bonds.

#### Molecular dynamic simulation

**Figure 4.**
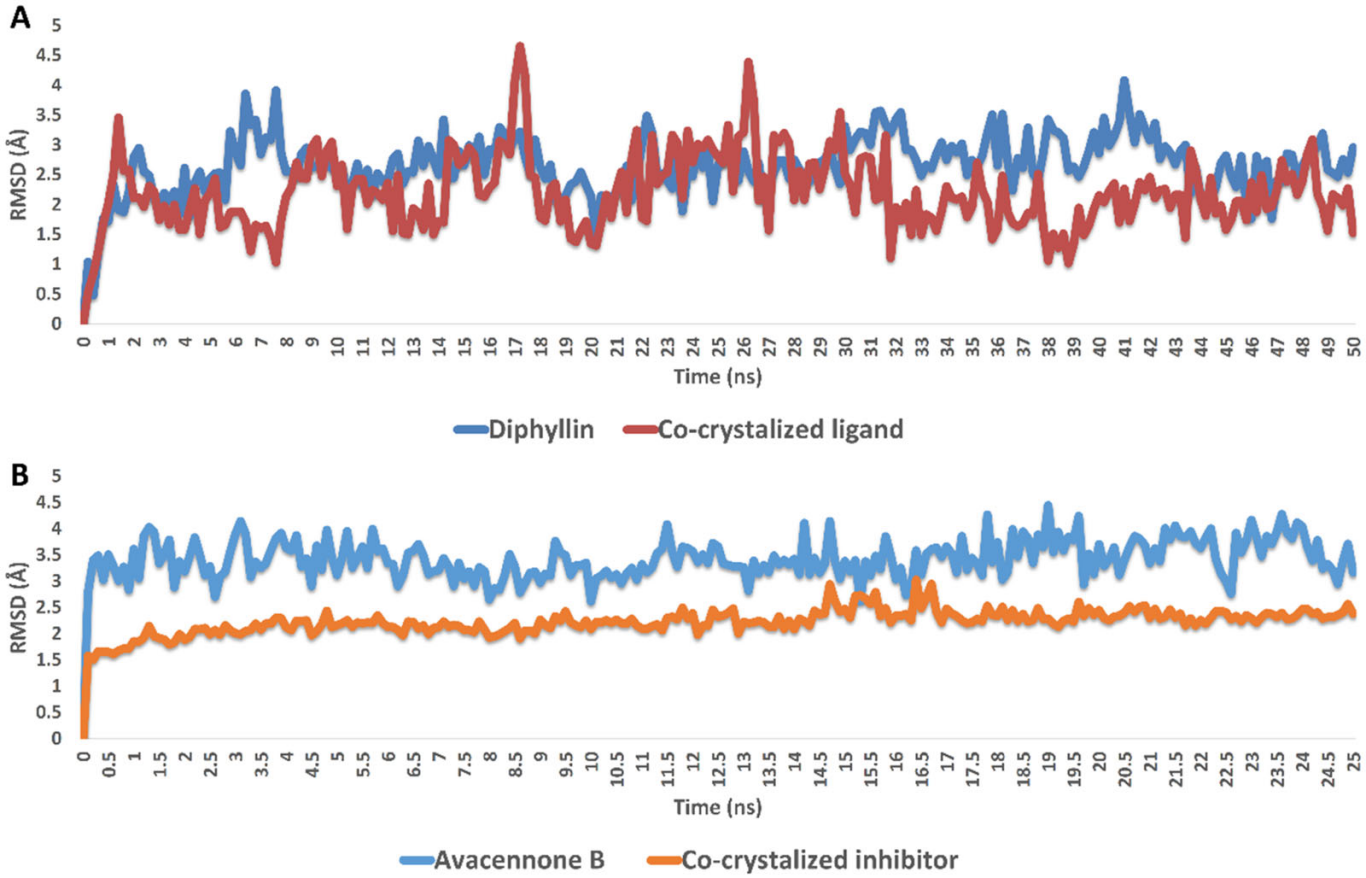
RMSDs of diphyllin and avacennone B inside the active sites of rhodesain and farnesyl diphosphate synthase, respectively, over 50 ns of MDS.

The binding free energy calculation (Δ*G*_binding_) and molecular dynamic simulation were carried out to further validate the inverse docking results. As shown in Figure 4, both diphyllin and avacennone B remained stable inside the binding pocket of rhodesain farnesyl diphosphate synthase over 50 ns of MDS, where their average deviations (average RMSD) from the initial binding pose were within acceptable values (average RMSD = 2.23 and 2.85 Å, respectively). Accordingly, the estimated absolute binding free energy of both compounds with rhodesain and farnesyl diphosphate synthase was comparable with that of the co-crystalized inhibitor (Δ*G*_binding_ = −8.23, −7.65 kcal/mol, Δ*G*_binding_ of the co-crystalized inhibitors = −8.34 and 8.45 kcal/mol, respectively).

**Figure 5.**
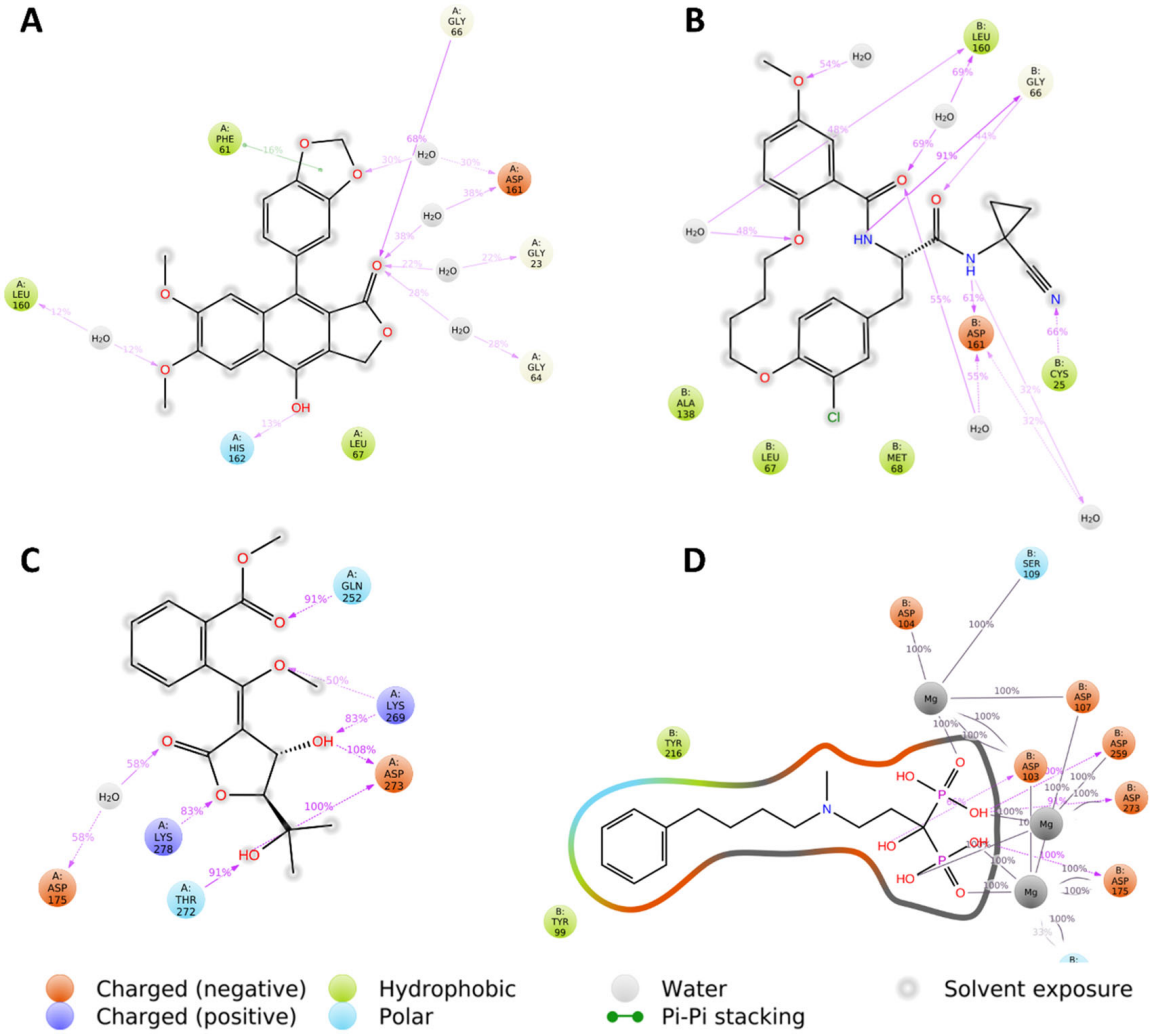
Protein-ligand contacts inside the rhodesain’ and farnesyl diphosphate synthase’s binding sites over 50 ns of MDS: (A–D) diphyllin and avacennone B alongside the corresponding co-crystalized ligands, respectively.

Regarding protein–ligand interactions during the simulation, diphyllin was able to establish H-bonds and water bridges with GLY-23, GLY-64, GLY-66, LEU-160, ASP-161, and HIS-162, together with hydrophobic interactions with PHE-61 and LEU-67 inside rhodesain’s active site (Figure 5). Similarly, avacennone B established multiple H-bonds and water bridges with ASP-175, GLN-252, LYS-269, THR-272, ASP-273, and LYS-278 inside farnesyl diphosphate synthase’s active site (Figure 5).

#### Pharmacokinetic and toxicity profiling

**Figure 6.**
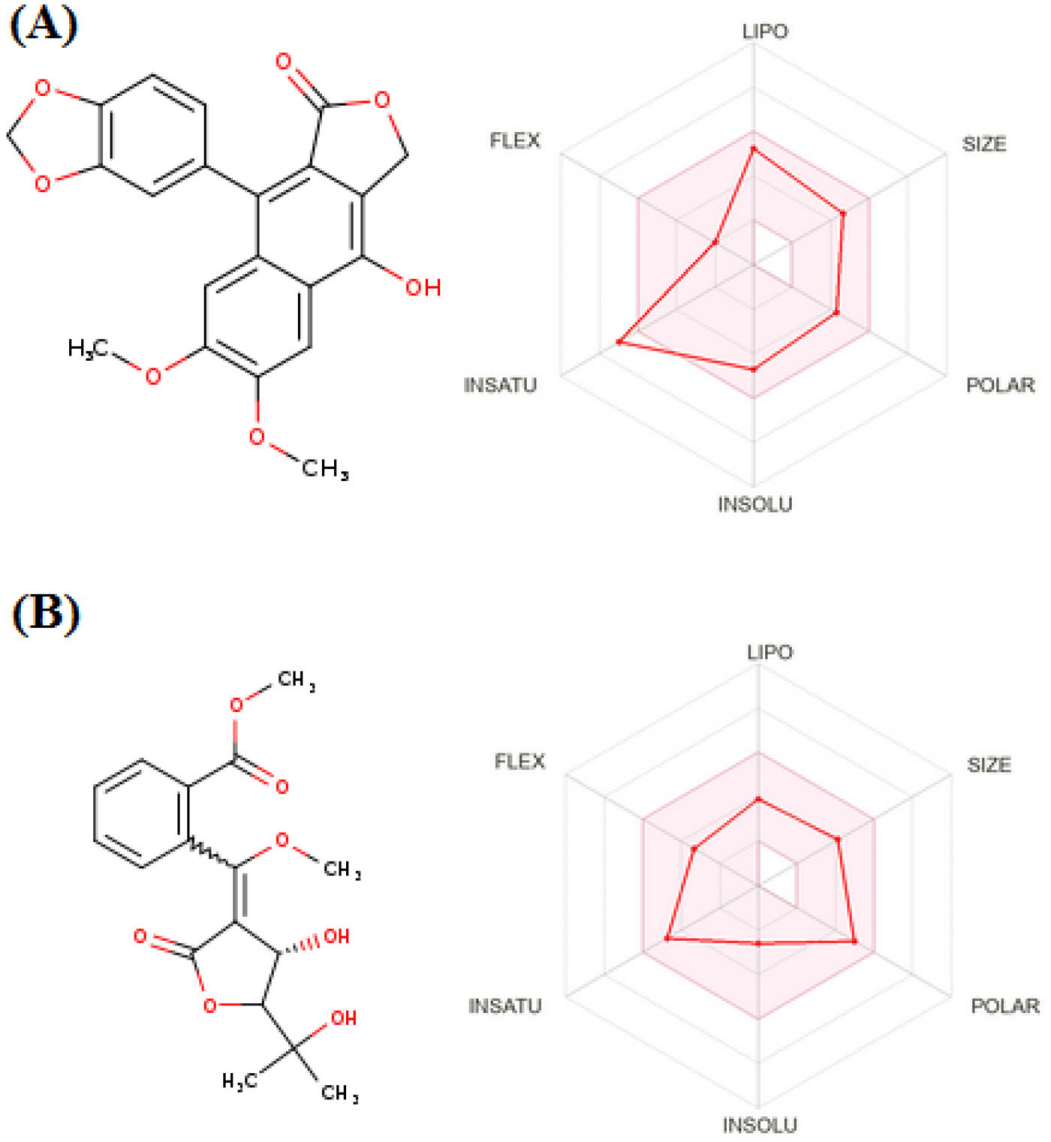
*In-silico* predicted pharmacokinetic properties of diphyllin (A) and avacennone B (B).

It is well established that drug candidate should have both acceptable pharmacological, pharmacokinetic and safety measures^54^^,^^55^. Accordingly, the ADMET profiles of diphyllin and avacennone B were calculated using SWISS ADME and PRO-ToX-II. In general, both compounds showed high degree of absorption from the gastrointestinal tract (GIT). This is attributed to the ability of both compounds to fulfil the required physicochemical properties for optimum absorption. As demonstrated by the properties radar chart, both the compounds had the desired values of all the properties (size, polarity, lipophilicity, flexibility, solubility, and saturation) with only exception for the saturation of diphyllin (Figure 6). This make both the compounds an excellent choice for oral use. Moreover, it is very important to get insights in the metabolic behaviour of both the compounds. Diphyllin was found to inhibit various isoforms of cytochrome enzymes such as CYP1A2, CYP2C19, CYP2C9, CYP2D6, and CYP3A4. On the other hand, avacennone B had no effect on any the previously mentioned cytochrome isoforms and thus it could be used safely with other drugs with no need for dose adjustment. A worthy note, is that both compounds had no violation any of the Druglikeness rules (Lipinski, viber, Muegee, ghose, veber, and egan) making them excellent drug candidates for future optimisation. Finally, both compounds have no records in pan interference assays (PAINS) giving rise to their potential high safety margin. We could not get any information about the toxicity profile of both compounds, however they have been reported in a number of previous *in vivo* studies^56^^,^^57^. Hence, to argument our safety claims, the toxicity of both the compounds were predicted by calculating the LD_50_ using PRO-ToX-II. Interestingly, diphyllin, and avacennone B had LD_50_ of 2100 mg/kg and 1130 mg/kg ensuring their safety margins.

## Conclusion

The present study spotted the effectiveness of metabolites identified by LC-HRESIMS for the chemical analysis of medicinal plants. Concurrently, the methanol extract of *T. grandifolia* showed potent anti-trypanosomal activity. Two of the dereplicated molecules in the plant extract (i.e. diphyllin and avacennone B) were identified as potential targets for rhodesain and farnesyl diphosphate synthase according to an *in silico* analysis that included inverse docking, molecular dynamic simulation, and absolute binding free energy. This work evoked the potential of *T. grandifolia* as a new prospective source of bioactive compounds for the management of trypanosomiasis.

## Supplementary Material

Supplemental Material
