# Supplementary material for: Metabolomic profile, anti-trypanosomal potential and molecular docking studies of Thunbergia grandifolia
Source: J Enzyme Inhib Med Chem. 2023 Apr 20;38(1):2199950. doi: 10.1080/14756366.2023.2199950 (PMC10120545; doi:10.1080/14756366.2023.2199950)
Supplement: Supplemental Material [file IENZ_A_2199950_SM1897.pdf]

Supplementary Material

Metabolomic Profile, Anti-Trypanosomal Potential and Molecular Docking Studies of *Thunbergia grandifolia* (Acanthaceae)

(a)

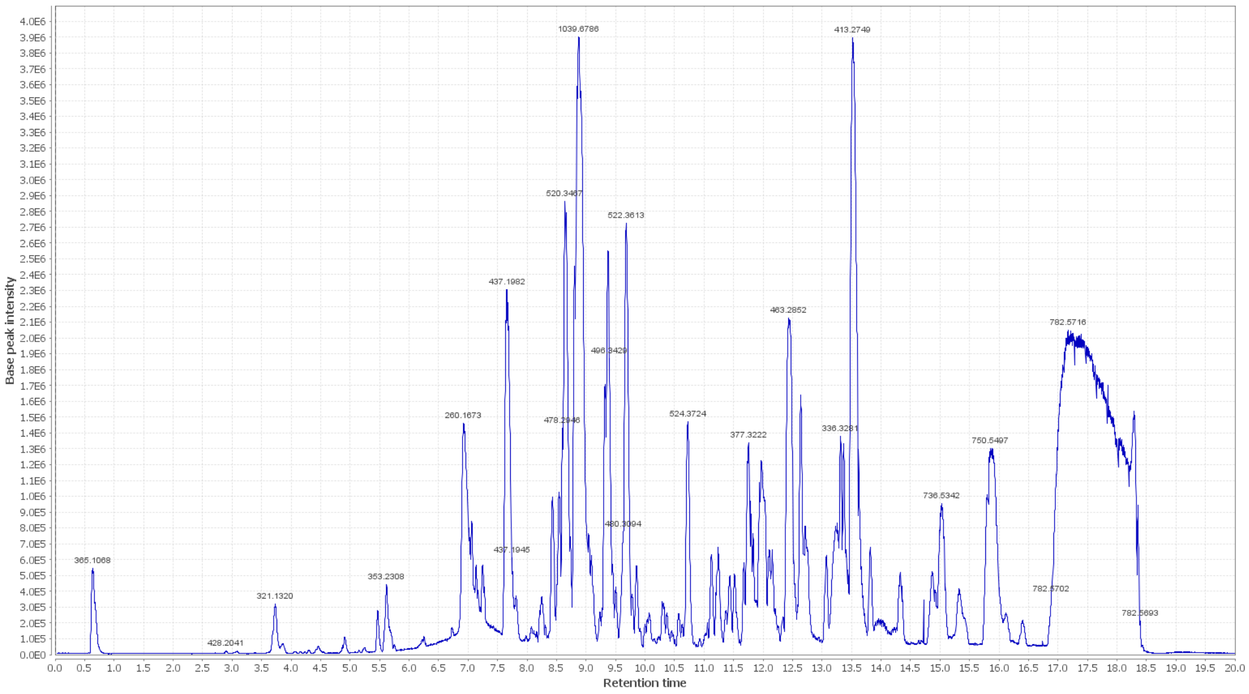

(b)

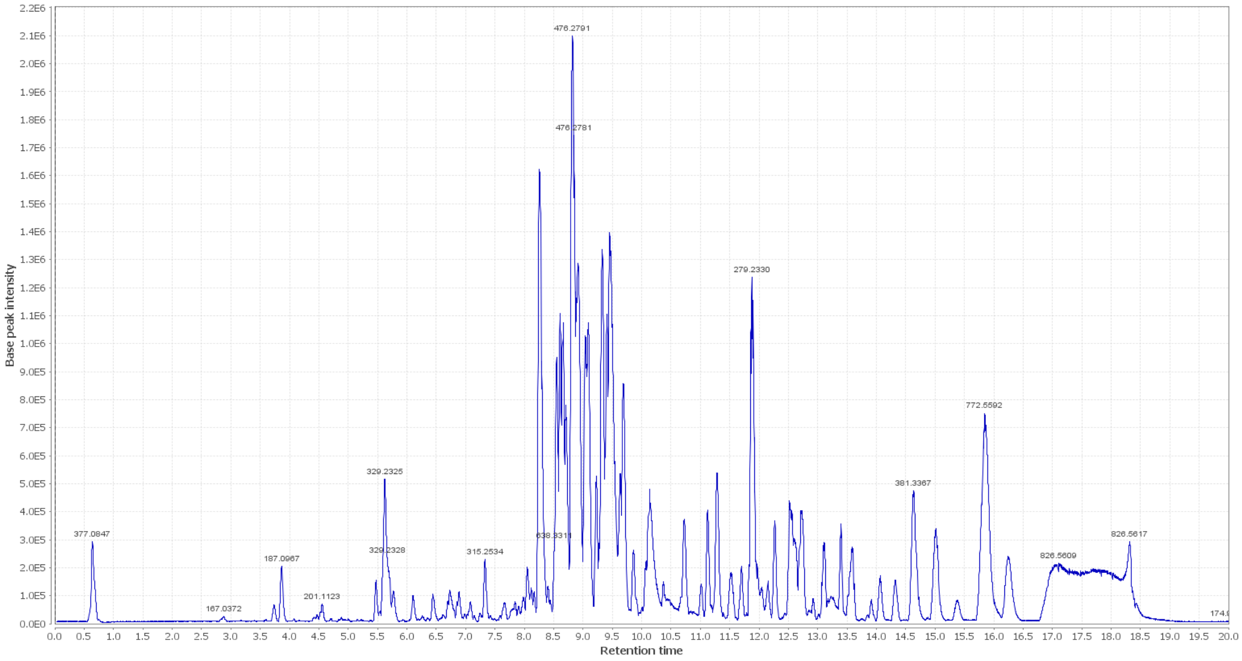

Figure S1. TIC of the methanol extract of *T. grandifolia*: (a) positive ion mode (b) negative ion mode.

Methods

Determination of the protein targets by inverse docking

Characterization of the biological target for a given drug molecule is a challenging task, however, the continuous development in the field of molecular modeling and virtual screening has significantly facilitated this process. Many online target identification search engines are currently available, and their search protocols are either ligand-based or structural based.

The potential protein targets for the studied extract were proposed by subjecting all of these compounds to inverse docking against all proteins hosted in the Protein Data Bank (PDB; https://www.rcsb.org/; accessed on 15 May 2021). The idTarget platform (http://idtarget.rcas.sinica.edu.tw/; accessed on 18 May 2022) was used for this task. This structure-based screening software applies a unique docking approach called divide-and-conquer docking that adaptively builds small overlapping grids to make the searching space on the protein surfaces more constrained. Hence, it can run a huge number of accurate docking experiments in a much-reduced time [1]. The retrieved results were obtained as a list of binding affinity scores arranged from the highest negative to the lowest. We set a binding affinity score of −9 kcal/mol as a cut-off value to select the best targets for each compound identified in the studied extract.

2. Molecular Dynamics Simulation

Desmond v. 2.2 software was used for performing MDS experiments [4–6]. This software applies the OPLS force field. Protein systems were built using the System Builder option, where the protein structure was embedded in an orthorhombic box of TIP3P water together with 0.15 M Na^+ and Cl^- ions in 20 Å solvent buffer. Afterward, the prepared

systems were energy minimized and equilibrated for 10 ns. Desmond software automatically parameterizes inputted ligands during the system building step according to the OPLS force field. For simulations performed by NAMD [7], the parameters and topologies of the compounds were calculated either using the Charmm27 force field with the online software Ligand Reader and Modeler (http://www.charmm-gui. org/?doc=input/ligandrm, accessed on 16 April 2021) [8] or using the VMD plugin Force Field Toolkit (ffTK). Afterward, the generated parameters and topology files were loaded to VMD to readily read the protein–ligand complexes without errors and then conduct the simulation step.

3. Binding Free Energy Calculations

Binding free energy calculations (ΔG) were performed using the free energy perturbation (FEP) method [7]. This method was described in detail in the recent article by Kim and coworkers [7]. Briefly, this method calculates the binding free energy $\Delta G_{\text{binding}}$ according to the following equation: $\Delta G_{\text{binding}} = \Delta G_{\text{Complex}} - \Delta G_{\text{Ligand}}$. The value of each ΔG is estimated from a separate simulation using NAMD software. Interestingly, all input files required for simulation by NAMD can be papered by using the online website CharmmGUI (https://charmm-gui.org/?doc=input/afes.abinding, accessed on 18 May 2021). Subsequently, we can use these files in NAMD to produce the required simulations using the FEP calculation function in NAMD. The equilibration was achieved in the NPT ensemble at 300 K and 1 atm (1.01325 bar) with Langevin piston pressure (for “Complex” and “Ligand”) in the presence of the TIP3P water model. Then, 10 ns FEP simulations were performed for each compound, and the last 5 ns of the free energy values was measured for the final free energy values [7]. Finally, the generated trajectories were visualized and analyzed using VMD software. It worth noting that Ngo and co-workers in their recent

benchmarking study found that the FEP method of determination of ΔG was the most accurate method in terms of predicting M^{Pro} inhibitors [7].

References

1. Wang, J.C.; Chu, P.Y.; Chen, C.M.; Lin, J.H. idTarget: A web server for identifying protein targets of small chemical molecules with robust scoring functions and a divide-and-conquer docking approach. Nucleic Acids Res. 2012, 40, W393–W399.
2. Yelshanskaya, M. V., Singh, A. K., Sampson, J. M., Narangoda, C., Kurnikova, M., & Sobolevsky, A. I. (2016). Structural bases of noncompetitive inhibition of AMPA-subtype ionotropic glutamate receptors by antiepileptic drugs. *Neuron*, *91*(6), 1305-1315.
3. Pan, X., Li, Z., Jin, X., Zhao, Y., Huang, G., Huang, X., ... & Yan, N. (2021). Comparative structural analysis of human Nav1. 1 and Nav1. 5 reveals mutational hotspots for sodium channelopathies. *Proceedings of the National Academy of Sciences*, *118*(11).
4. Bowers, K.J.; Chow, D.E.; Xu, H.; Dror, R.O.; Eastwood, M.P.; Gregersen, B.A.; Klepeis, J.L.; Kolossvary, I.; Moraes, M.A.; Sacerdoti, F.D.; et al. Scalable algorithms for molecular dynamics simulations on commodity clusters. In Proceedings of the SC'06: Proceedings of the 2006 ACM/IEEE Conference on Supercomputing, Tampa, FL, USA, 11–17 November 2006; IEEE: New York, NY, USA, 2006; p. 43.
5. Release, S. 3: Desmond Molecular Dynamics System, DE Shaw Research, New York, NY, 2017; Maestro-Desmond Interoperability Tools, Schrödinger: New York, NY, USA, 2017.
   7. Schrodinger LLC. Maestro, Version 9.0; Schrodinger LLC: New York, NY, USA, 2009.
6. Phillips, J.C.; Braun, R.; Wang, W.; Gumbart, J.; Tajkhorshid, E.; Villa, E.; Chipot, C.; Skeel, R.D.; Kalé, L.; Schulten, K. Scalable molecular dynamics with NAMD. J. Comput. Chem. 2005, 26, 1781–1802.
7. Kim, S.; Oshima, H.; Zhang, H.; Kern, N.R.; Re, S.; Lee, J.; Rous, B.; Sugita, Y.; Jiang, W.; Im, W. CHARMM-GUI free energy calculator for absolute and relative ligand solvation and binding free energy simulations. J. Chem. Theory Comput. 2020, 16, 7207–7218.
8. Ngo, S.T.; Tam, N.M.; Quan, P.M.; Nguyen, T.H. Benchmark of Popular Free Energy Approaches Revealing the Inhibitors Binding to SARS-CoV-2 Mpro. J. Chem. Inf. Model. 2021, 61, 2302–2312.
